# Beyond survival: the imperative, innovation, and implementation of long-term intensive care unit follow-up

**DOI:** 10.62675/2965-2774.20260479

**Published:** 2026-09-15

**Authors:** José Mário Meira Teles, João Gabriel Rosa Ramos, David Pilcher

**Affiliations:** 1 Hospital Geral Ernesto Simões Filho Salvador BA Brazil Intensive Care Unit, Hospital Geral Ernesto Simões Filho - Salvador (BA), Brazil.; 2 Clínica Florence Salvador BA Brazil Clínica Florence - Salvador (BA), Brazil.; 3 Department of Intensive Care, Alfred Hospital, Bayside Health Melbourne Australia Department of Intensive Care, Alfred Hospital, Bayside Health - Melbourne, Australia.; 4 Australian and New Zealand Intensive Care Society Centre for Outcome and Resource Evaluation Melbourne Australia Centre for Outcome and Resource Evaluation, Australian and New Zealand Intensive Care Society - Melbourne, Australia.

## THE "LEGACY" OF CRITICAL ILLNESS

This viewpoint reflects the authors’ interpretative perspective, informed by current evidence and clinical experience. For decades, intensive care success was judged mainly by intensive care unit (ICU) or hospital mortality. If a patient survived to discharge, the job was considered complete. That definition is no longer adequate. Millions of ICU survivors leave the hospital carrying a "legacy" of critical illness, frequently expressed as post-intensive care syndrome (PICS). This syndrome encompasses new or worsening physical disability, cognitive impairment, and mental health symptoms that can affect long-term survival, readmissions, social functioning, and quality of life.^(1)^ Notably, PICS occurs across heterogeneous populations and is not limited to survivors of invasive mechanical ventilation.

Despite the growing recognition of this burden, post-ICU follow-up remains fragmented. Recent syntheses highlight a widely shared moral imperative to support survivors, yet divergences on "best practice" models, limited evidence, and persistent practical barriers remain.^(2)^ This results in significant heterogeneity in care delivery. The paradox is both ethical and pragmatic: we deploy advanced technology to save lives in the ICU, yet often leave survivors and families to navigate a complex recovery without a map. Intensive care unit performance cannot be framed solely by in-hospital outcomes. Long-term follow-up is a core element of the continuum of critical care, essential both to measure patient-centered outcomes and to mitigate PICS sequelae. The challenge lies in operationalizing the response amidst resource constraints.

## POSSIBLE SOLUTIONS: BRIDGING THE GAP WITH TECHNOLOGY

A substantial proportion of ICU survivors have unmet post-discharge needs due to limited structured services and multidisciplinary access.^(3–5)^ Follow-up models and referral pathways vary widely across settings. This drives inconsistent survivorship care and persistent recovery gaps. Scalable technology-enabled follow-up (telemedicine, automation, and remote monitoring) can expand access while maintaining clinical oversight. A traditional, face-to-face multidisciplinary clinic for every survivor is logistically and financially unrealistic for most centers.

A pragmatic solution may be a stratified hybrid model that integrates telehealth with automation and selective use of Artificial Intelligence (AI). Telehealth reduces geographic barriers for frail survivors and may improve attendance.^(2)^ While virtual ICU recovery clinics are feasible, current evidence remains emerging. Telehealth's immediate value lies in access, continuity, and early signal detection, while the field builds stronger comparative evidence and standardized evaluation benchmarks.

In addition to consultations, AI and automation can support risk stratification and longitudinal monitoring. Machine learning algorithms can analyze ICU-derived data such as *delirium* burden, sedation exposure, ventilation duration, and signs of functional recovery captured during the ICU stay, including the timing and progression of patient mobilization, to estimate risk in specific PICS domains. This enables precise follow-up, directing scarce resources like neurocognitive testing and physical therapy to those at highest risk. Furthermore, conversational AI and voicebots could perform routine screenings at 1, 3, and 6 months, alerting teams only when risk thresholds are exceeded. In this "human-in-the-loop" design, a single navigator could oversee large cohorts, reserving specialist physicians’ time for complex assessments requiring human judgment.

Innovation also extends to passive monitoring and data linkage.^(6–8)^ Automated surveys, remote mobility monitoring through wearables, and linkage to administrative or health-system datasets (e.g., disability benefits, pharmacy records, and national death registries) provide a comprehensive view of recovery beyond clinic walls. Telephone follow-up, in Australian trauma registries, can support targeted populations but may miss patients who are too busy or too unwell to respond. Interestingly, some systems have "flipped the approach" by using public health measures to reduce ICU admissions altogether, such as alcohol-reduction initiatives that lowered alcohol-related admissions and ICU readmissions.^(9)^ Future ICU admission can itself be a marker of success or failure of mental health interventions in some populations.^(10)^

Adoption of technology-enabled programs depends on cost transparency and payment feasibility. Tele-rehabilitation and tele-monitoring should not be assumed to be inherently cost-saving; economic conclusions vary depending on the analytic perspective (health system *versus* societal) and the inclusion of infrastructure and downstream utilization costs. Payer negotiations are most persuasive when supported by pragmatic data combining patient-reported outcomes, functional recovery, and healthcare utilization. Ideally, cost outcomes should include both the health system (readmissions, emergency visits) and the patient/caregiver perspective (out-of-pocket costs, time burden).

## A ROADMAP FOR IMPLEMENTATION

To move from theory to practice, leaders should adopt a stepwise, pragmatic roadmap:

1. **Define the target population (screening):** do not attempt to follow every survivor initially. Establish local "high-risk" criteria, such as survivors ventilated for > 48 hours or an ICU stay >72 hours,^(11)^ or documented delirium. Screening should start at ICU discharge and be completed at hospital discharge. Survivors meeting these criteria should leave the hospital with a scheduled appointment or a documented plan. Tailor strategies to specific needs: working-age adults may benefit from return-to-work outcomes, while remote patients require telehealth-first pathways.^(12)^
2. **Engage stakeholders early:** a follow-up service cannot function in a silo. Success depends on the "handshake" with primary care. The clinic's aim should be to identify PICS domains, initiate early interventions, and recommend pathways rather than permanently replacing primary care. Define local "success" metrics and establish referral pathways to community rehabilitation and mental health services before the first patient is seen.
3. **Start small (the pilot):** launch a limited session, such as a half-day per week, using existing staff and teleconsultations. Use the pilot to build workflow, test engagement, and generate local data. Return on investment should be measured as avoided costs and improved patient-reported outcomes. Incorporating short-cycle pragmatic randomized evaluations embedded in implementation can generate timely causal evidence on effectiveness.^(13)^
4. **Standardize assessments:** use validated and comparable tools to ensure data reliability.^(14)^ For example, use the modified Barthel Index for functional status, the EuroQol 5-Dimension (EQ-5D) for quality of life, the HADS for anxiety/depression, and the MoCA for cognition (Table 1). Make results visible within the institution. Follow-up data become the currency for engaging stakeholders and advocating for sustainable funding.
5. **Iterate and scale:** once the workflow is refined, expand the inclusion criteria. The ultimate goal is a culture shift where the ICU discharge summary is not the final chapter, but the introduction to a supported recovery.

**Table 1:** Practical recommendations for standardized assessment in post-intensive care unit follow-up

Domain	Recommended tool	What it measures	Typical time	Scoring and common interpretation	Practical notes
Functional status	Modified Barthel Index	Basic activities of daily living independence	5 - 10 minutes	Commonly 0 - 100 (higher = more independence), depending on the modified version used	Modified Barthel has multiple variants. State exactly which version and scoring anchors you used to avoid comparability problems
Health-related quality of life	EQ-5D (3L or 5L, plus EQ-VAS)	Generic HRQoL across 5 dimensions + self-rated health VAS	2 - 5 minutes	Produces a health profile and index value (using country value sets) plus VAS 0 - 100	Registration/licensing required; non-commercial use is free after registration per EQ policy
Anxiety/ depression	HADS	Anxiety (7 items) and depression (7 items) symptom burden	5 - 7 minutes	Each subscale 0 - 21. A common approach: 0 - 7 normal, 8 - 10 borderline, ≥ 11 probable case	HADS is copyrighted, and licensing may apply depending on use/setting
Cognition	MoCA	Global cognitive screening (attention, executive function, memory, language, visuospatial)	10 minutes	Total 0 - 30. Many studies use < 26 as an impairment screen, though optimal cutoffs can vary by population/ education	Certification is optional when MoCA is used solely for screening, triage, or referral based on the total score

EQ-5D - EuroQol 5-Dimension; L - levels per dimension; VAS - visual analog scale; HRQoL - health-related quality of life; HADS - Hospital Anxiety and Depression Scale; MoCA - Montreal Cognitive Assessment.

Source: adapted from: Bornemann-Cimenti H, Lang J, Hammer S, Lang-Illievich K, Labenbacher S, Neuwersch-Sommeregger S, et al. Consensus-based recommendations for assessing post-intensive care syndrome: a systematic review. J Clin Med. 2025;14(10):3595.^(14)^

## Data Availability

The contents underlying the research text are included in the manuscript.
